# Minimal Clinically Important Differences (MCID) for the Functional Assessment of Chronic Illness Therapy Fatigue Scale in Patients with Systemic Sclerosis

**DOI:** 10.3390/ijerph20010771

**Published:** 2022-12-31

**Authors:** Franciska Kiss, Nelli Farkas, Gabriella Nagy, Tünde Minier, Gábor Kumánovics, Réka Faludi, László Czirják, Cecília Varjú

**Affiliations:** 1 Department of Rheumatology and Immunology, Medical School, University of Pécs, H-7632 Pécs, Hungary; 2 Institute of Bioanalysis, Medical School, University of Pécs, H-7624 Pécs, Hungary; 3 Heart Institute, Medical School, University of Pécs, H-7624 Pécs, Hungary

**Keywords:** systemic sclerosis, fatigue, health-related quality of life, minimal clinically important difference, functional assessment of chronic illness therapy, fatigue scale

## Abstract

(1) Background: Systemic sclerosis (SSc) is characterized by significant fatigue, causing diminished quality of life (QoL). The aim of this study was to examine fatigue levels and their associations with clinical factors and determine the minimal clinically important difference (MCID) value for the Functional Assessment of Chronic Illness Therapy Fatigue Scale (FACIT-FS). (2) Methods: A total of 160 SSc patients and 62 individuals without SSc were followed-up over a 12-month period by measuring the FACIT-FS and the Visual Analogue Scale and the Short Form 36 Vitality Score analyzing changes in exhaustion. (3) Results: Fatigue was strongly correlated with HRQoL, level of pain, emotional disorders, physical capability and functionality. The MCID values for FACIT-FS were calculated as −3 for deterioration and +4 for improvement after a 12-month follow-up. The predictors of improvement of fatigue from baseline parameters were the significant disease activity, the patients’ poorer functionality and the short disease duration. Patients with scleroderma-related interstitial lung disease at baseline had approximately tripled risks for worsening fatigue. The independent influential factors regarding the changing of FACIT-FS were improving or worsening in the same direction in reference to physical condition, gastrointestinal and emotional factors. (4) Conclusions: Fatigue is a multi-dimensional symptom, which is strongly correlated to HRQoL. MCID values of FACIT-FS can be useful tools in monitoring the changes of HRQoL in clinical trials and in daily practice among patients with SSc.

## 1. Introduction

Systemic Sclerosis (SSc) is a connective tissue disease characterizing vasculopathy, inflammation, and fibroblast dysfunction, which affects the skin, the musculoskeletal system and internal organs. It is a rare disease, with a prevalence of 7.2–33.9 per 100,000 individuals reported throughout Europe. The 10-year survival rates are 65–73% [1]. This study was conducted in a tertiary care centrum in Hungary, in which 350–400 patients were treated with SSc [2]. While most of the endeavors aim to prevent end-stage organ damage, less attention focuses on symptoms, which bears a detrimental effect on health-related quality of life (HRQoL), including abnormal fatigue [3].

Chronic fatigue is a common companion of most of systemic autoimmune diseases, such as SSc. In a study incorporating 463 scleroderma patients, fatigue was the most frequently reported symptom (89%), followed by Raynaud’s phenomenon, hand stiffness, and joint pain [4]. In contrast to fatigue often perceived by individuals without SSc, abnormal, chronic fatigue has a consistently impairing effect upon patients’ life for more than 6 months and it is not thoroughly alleviated by rest. Since it is a less elusive, relatively subjective symptom, it is often neglected during medical examinations [3]. Although patients often report fatigue as the most problematic symptom, due to the limits of their daily routine [5]. It has a prominent impact upon employability and on HRQoL. In a published study [6], patients afflicted with higher fatigue were more likely to be less productive at the workplace, and unable to do household duties, while another examination conducted with 57 scleroderma patients of working age [7], employment status showed the strongest correlations with pain, fatigue, hand function deterioration and digital ulcers. Fatigue correlated with HRQoL, and it was also a predictor of HRQoL deterioration in 337 scleroderma patients, according to Hudson et al. [8].

In SSc, fatigue is likely linked to inflammatory and immune-mediated reactions’ impact on the central nervous system or may be a result of other SSc-related organ damage [9]. Several internal organ involvements, such as pulmonary, renal and cardiac parameters, correlated with fatigue in a longitudinal study, which also demonstrated loss of functionality, especially hand function deterioration associated with increasing fatigue [10]. There is supportive evidence in which GI, muscle and joint manifestations and pain may also be contributing factors [11]. When considering the psychological aspects, depression and exhaustion symptoms often overlap, moreover, individuals suffering from fatigue were two times more likely to also develop depression, according to a large study [12]. In 566 scleroderma patients, the score of two depression scales: The Center for Epidemiologic Studies Depression Scale and the Patient Health Questionnaire Depression Scale were highly linked with fatigue levels [13], while others demonstrated its connection with smaller social network size and diversity [14]. Despite this information emphasizing the complexity of this symptom, very few longitudinal studies were performed to examine factors leading to the worsening of fatigue, and fatigue’s impact on patients’ future lives. Factors, which may help improve fatigue symptoms might be suitable targets for interventions toward improving and preventing fatigue-related problems.

The aim of this study is to assess the severity of this symptom among the various scleroderma subgroups and non-SSc-suffering controls while identifying fatigue-predicting factors which are related to organ manifestations and demographic, psychosocial characteristics. Furthermore, it was an objective to follow up changes in fatigue severity during the disease course, detect the factors predisposing the impairment of fatigue, and reveal those factors changing in parallel with fatigue levels, since only a few longitudinal studies are currently published. There were also only a few interventional studies, and this information may prove towards identifying new intervention strategies. Additionally, it was a goal to determine the Minimal clinically important difference (MCID) values regarding the Functional Assessment of Chronic Illness Therapy Fatigue Scale (FACIT-FS). Using MCID values during these kinds of studies may help in following patients in a more precise way.

## 2. Patients and Methods

In this study, 160 consecutive cases with SSc were included from the Department of Rheumatology and Immunology, Medical School of the University of Pécs. Data collection incorporated a baseline study and a 12-month follow-up, comprising a control group of 62 volunteers without SSc, matched in age and gender. Data were successfully obtained following an average of 12 months as a follow-up examination, from 144 of the 160 SSc patients at baseline. Of the patients, 11 patients’ data were missing during the second examination, 4 among them succumbed during the follow-up period, 7 were missing from the patient care due to unknown reasons and 5 more cases were invalidated due to questionnaire completion errors. All participants gave their informed written consent to the study, which was conducted according to the Declaration of Helsinki, and approved by the Ethics Committee of National Public Health Centre Institutional Committee of Science and Research Ethics (39672-6/2020/EÜIG, 2020.09.03., Budapest, Hungary).

### 2.1. Examination of the Degree of Fatigue

To assess fatigue severity, the Functional Assessment of Chronic Illness Therapy-Fatigue Scale (FACIT-FS) and the Vitality subscale of the Short Form Health Survey 36 (SF36-VT) were used [15]. Participants also valued fatigue perceived by individually using a 0–100 mm visual analogue scale (VAS Fatigue) [15]. The FACIT-FS is a part of a complex HRQoL measuring questionnaire, the Functional Assessment of Chronic Illness Therapy-F (FACIT-F) [16]. The FACIT-FS contains 13 statements referencing fatigue and its impact on the subject’s life quality. Scoring for every statement ranges from 0 to 4 (not at all—very much), with a maximum score of 52, in which 52 indicates the potentially best condition and 0 is the worst. A subscale from the SF36 questionnaire, SF36-VT, is a general measure of energy and fatigue, is formed from the entire questionnaire’s 4 questions (9a, 9e, 9g, 9i), in which 9a and 9e inquire regarding pep and energy, while 9g and 9i represent exhaustion and weariness. The maximum score is 100, in which the lower scores indicate higher fatigue levels. In VAS Fatigue, higher scores are associated with worse conditions [15].

### 2.2. HRQoL and General Health Evaluations

To measure HRQol, the FACIT-F [17] and the SF36 [18] questionnaires were used. Both questionnaires have several dimensions according to the aspects of HRQoL, of which the following were used in the current study: mental well-being was evaluated using the SF36 Mental Component Summary (SF36-MCS), despair, hopelessness and anxiety symptoms were examined using FACIT Emotional Well-being (FACIT-EWB) questionnaires. In contrasting patients with or without anxiety and depression symptoms, the related domain of the Euro Quality of Life-5 Dimension (EQ-5D) questionnaire was used [19]. The SF36 Physical Component Summary (SF36-PCS) was used to accumulate information regarding physical conditions with a self-administered modality.

To measure general health and functionality the Health Assessment Questionnaire Disability Index (HAQ-DI) [20] and Cochin Hand Function Scale (CHFS) [21] were used. 

Muscle function was assessed by a physiotherapist in 8 regions using the Manual Muscle Testing-8 (MMT-8) procedure [22]. The 6 min walk test (6 MWT) [23] was performed to measure aerobic capacity and physical performance. Pinch strength was measured by a B&L Engineering Pinch Gauge on the dominant hand of participants.

### 2.3. Patients’ Social and Demographical Characteristics

Data regarding past medical history, demographic and social characteristics were collected through a comprehensive patient interview. The data contains marital status, level of education, employment status and tobacco use habits. Cognitive functions were assessed using the Mini Mental State Examination (MMSE) [24].

### 2.4. Clinical and Laboratory Findings

Scleroderma characteristic manifestations, such as puffy fingers, joint contractures, digital ulceration and subcutaneous calcinosis were evaluated, as well as the modified Rodnan Skin Score (MRSS) [25], in which >14 points indicate moderate or severe activity among diffuse cutaneous SSc (dcSSc) patients [26]. Distinctively, tenderness and swelling in 28 joints were evaluated to calculate Disease Activity Score-28 (DAS-28) score [27]. 

Laboratory findings, particularly erythrocyte sedimentation rate (ESR), hemoglobin (Hgb) and albumin levels were recorded. Autoantibody assays were carried out, and participants were grouped based on their autoantibody profile. The presence of anti-nuclear antibodies (ANA), extractable nuclear antigens (ENA), anti-topoisomerase I (anti-Topo I), anti-centromere (ACA) and anti-RNA polymerase III (anti-RNAP3) (RP11 and RP155) antibodies in the serum sample of patients were measured using commercial ELISA (ORG 546 and 633 Orgentec Diagnostika, Mainz, Germany) and immunoblot kits (Euroline Systemic Sclerosis Profile, Euroimmun, Lübeck, Germany) according to the manufacturer’s protocol. 

Pulmonary involvement was diagnosed, if lung fibrosis was proven with High-resolution computed tomography or X-ray, or/and forced vital capacity (FVC) levels were predictably less than 80%, or diffusion capacity (DLCO) fell below 70% of the averaged normal [28]. 

The gastrointestinal (GI) symptom evaluation was based on the University of California Los Angeles Scleroderma Clinical Trials Consortium Gastrointestinal Tract 2.0 (UCLA-GIT 2.0) score. We defined GI involvement as ≥0.5 points [29]. 

Cardiac manifestation was characterized by the presence of reduced left ventricular ejection fraction (LVEF < 50%), relaxation disorder, arrhythmias and conduction disturbances, or pericardial fluid. Calculating left ventricular mass (LVM) was based on the Devereux formula and then indexed for body surface area (LVM index) [30]. Left ventricular diastolic function was assessed by an experienced cardiologist (RF) in consideration of mitral inflow pattern (E/A ratio), left ventricular mass index (LVMI), left atrial size and calculated right ventricular systolic pressure [31]. 

Based on clinical and laboratory findings, the revised European Scleroderma Trials and Research Group Activity Index (rEUSTAR-AI) was calculated for each patient, in which case the active disease was characterized by ≥2.5 points [32].

### 2.5. Statistical Analysis

Data analyses were implemented using IBM SPSS for Windows (version 25.0; IBM Corp., Armonk, NY, USA) and the R statistical software (version 4.2.1; packages: “randomForest” [33]; “wmwpow” [34]; R Core Team, Vienna, Austria). Normally distributed continuous variables were expressed as a mean ± standard deviation, data with a non-normal distribution, the median was used with the first and third quartiles. Categorical data were described as frequencies and percentages. Independent sample *T*-test and Mann–Whitney U test were used to compare baseline characteristics in continuous data and the Chi-square test regarding categorical variables. Wilcoxon signed-rank test was used for analyzing the differences of test-results between baseline and at follow-up time points of the study. Correlations between baseline variables were assessed with Spearman’s rank correlation. To determine MCID for FACIT-FS, the Visual Analogue Scale (VAS) in reference to fatigue and the Mental Component Score of the SF36 questionnaire (SF36-MCS) were used as anchors in support of this process, since the MCID of these patient-reported outcome measures (PROM) have been previously defined among SSc patients. ROC curve analyses were performed to examine the discriminative performance of FACIT-FS. Accordingly, specificity and sensitivity were calculated for the determined MCID values. A machine learning algorithm referred to Random Forest (RF) analysis was used to detect potential predictors of fatigue changes. We built 1000 RF, in each “forest” 500 trees were grown to produce the predictions. Each tree was grown using the conditional inference method, to avoid bias toward dependent predictors and overfitting [35]. The relative importance was determined by summarizing the results of the RFs for each of the predictors. Binary logistic regression analyses were administered to determine the independent predictors of fatigue improvement or worsening. In all statistical tests, a *p*-value < 0.05 was considered as significant.

## 3. Results

### 3.1. Result of the Baseline Examination

Table 1 depicts key demographics and clinical parameters from the 160 patients with SSc and 62 non-SSc-suffering controls. The proportion of females was similar between control group and SSc patients, with no significant difference in mean age. However, for almost all other parameters seen in Table 1, patients with scleroderma showed significantly worse results compared to non-SSc-suffering controls, regarding clinical parameters and physical symptoms.

Moodiness was more frequent among SSc patients, they showed more fatigue and poorer HRQoL. Less scleroderma patients were employed compared to non-SSc-suffering controls (Table 1). Other important scleroderma-related disorders are pulmonary arterial hypertension, which was confirmed by right heart catheterization in five patients, and scleroderma renal crisis, which occurred in two patients.

The correlations between the three fatigue measuring tests and clinical factors were examined, and the results are represented in Table 2. Levels of fatigue highly correlated with physical and mental/emotional well-being, however the degree of pain experienced by patients and the complaints associated with Raynaud’s phenomena also showed a strong correlation. Functional disability and hand function, the extent of GI complaints, muscle strength and the result of 6MWT also showed intense associations with exhaustion. Measures of fatigue were strongly associated with the results of HRQoL questionnaires. Moderate correlations were found between fatigue and age, cardiac E/A ratio and the results of the cognitive test, the MMSE. While all parameters mentioned above correlated with all three used fatigue measures, the rEUSTAR-AI showed mild correlation only with FACIT-FS. Interestingly, MRSS correlated positively with SF36-VT, and negatively with VAS Fatigue among dcSSc patients. In order to verify this relationship, the correlation examination was performed again, only with early dcSSc patients, in which case the skin score is important, and no correlations were found between the MRSS and the results of fatigue measures.

When comparing the subgroups of patients, in the case of gender, females suffered higher levels of fatigue based on FACIT-FS compared to males (*p* = 0.025), however, males were several years younger (57.2 ± 12.1 vs. 46.6 ± 12.2 *p* = 0.002). 

SSc patients were categorized according to the subtype of the disease (limited cutaneous SSc (lcSSc)-dcSSc). The average age and disease duration among lcSSc patients (59.9 ± 9.7 and 12.8 ± 8) were several years higher than patients with dcSSc (52.3 ± 14.6 and 8.7 ± 6.7) (*p* < 0.001 in both cases). Although muscle strength using MMT-8, and exercise capacity/physical performance measured by 6MWT were found to be worse in the lcSSc group (*p* = 0.002 and *p* = 0.036), fatigue tests showed similar results in the two subgroups (*p* = 0.092), including the results of the FACIT-F and SF36-PCS HRQoL questionnaires (*p* = 0.213). The only exception was the SF36-MCS questionnaire, in which case, the younger dcSSc patients, with shorter disease duration, achieved higher scores (*p* = 0.038).

Interestingly, when comparing early-phase and late-phase patients, there was no significant difference in FACIT- FS test results between the two subgroups (Table 3). Although, in the late disease duration group, functional disability using HAQ-DI and hand function deterioration using CHFS were more severe (*p* = 0.015 and *p* = 0.030). The physical component of the SF36 test and the 6MWT indicated poorer conditions among late-phase patients (*p* = 0.010 and *p* = 0.003).

Inexplicably, in 28 patients (17.8%), no scleroderma-related autoantibodies were detected. Only ANA was present in 39 cases (24.8%). Anti-Topo I was apparent in 41 (26.1%), ACA in 32 (20.4%), and Anti-RNAP3 in 17 (10.8%) cases. The remaining 3 patients had multiple autoantibodies therefore they were excluded from the following calculation. In comparing these patients based on autoantibodies, there was no significant difference in fatigue levels measured by the FACIT-FS (Figure S1. Supplementary Material) nor the other fatigue tests. 

Active SSc patients experienced more severe fatigue measured with FACIT-FS, when compared to non-active patients (Table 3). 

During the examination of patients according to the presence or absence of internal organ involvements, patients with GI manifestation had more severe fatigue, than patients without. Borderline significance was detected when examining the groups based on the absence or presence of pulmonary involvement. In the case of cardiac involvement, the difference based on FACIT-FS was not significant between the groups. 

Patients with more pain, and disabilities measured with HAQ-DI had significantly higher levels of fatigue. Fatigue was higher among the patients with anxiety and depression symptoms. Only 50 SSc patients were employed, and they had significantly lower fatigue when compared to unemployed individuals (*p* < 0.001), however, they proved to be younger (*p* < 0.001). Interestingly, 111 patients were of working age, among them 64 were employed, and their fatigue appeared to be still significantly milder (Table 3). Lastly, 67 patients lived alone, who were similar in age to the other 93 patients (*p* = 0.248), however, fatigue was more severe among the isolated based on the FACIT-FS (Table 3).

Sleeping disturbances were apparent in half of the patients, who reported significantly higher levels of exhaustion (Table 3).

### 3.2. Results of the Follow-Up

The correlation and the comparison examinations of the varied patient subgroups were repeated following 12 months, which yielded similar results as in the baseline. 

The median (Q1; Q3) values of FACIT-FS at baseline was 38.50 (29.25; 45.00) and 39.00 (28.5; 40.0) at follow-up. Overall, the results for the entire cohort did not tend to change significantly over the course of a year (*p* = 0.607). However, since fatigue levels did not change among the entire cohort, investigations were conducted on how to measure changes in fatigue at individual levels. 

**Figure 1:**
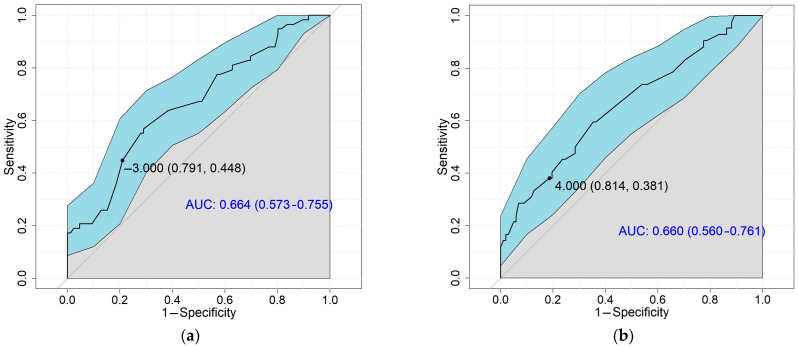

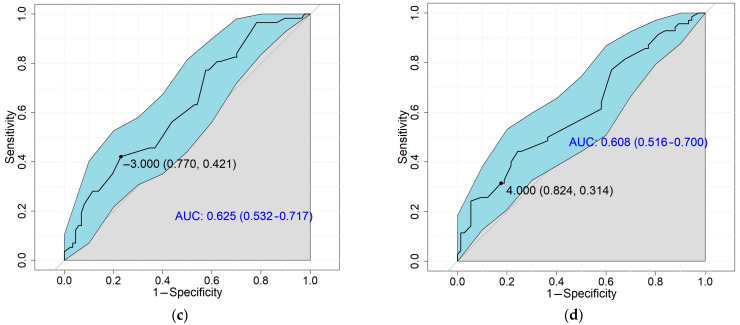
The discriminating ability of the Functional Assessment of Chronic Illness Therapy Fatigue Scale calculated with ROC curves, and sensitivity and specificity of the minimal clinically important difference values regarding the Visual Analogue Scale for Fatigue for (**a**) worsened, (**b**) improved; and the Short Form 36 Vitality scores for (**c**) worsened, (**d**) improved, based on data of the 144 followed patients with systemic sclerosis. ROC, receiver operating characteristic curve; AUC, Area Under the Curve.

The MCID values for FACIT-FS were determined, enabling patients to be independently classified, according to whether their fatigue worsened or improved over a 12-month period. Based on the VAS Fatigue, the MCID value for worsening fatigue in FACIT-FS was −3 points, and the same results were experienced regarding SF36-MCS. During ROC curve analysis, according to VAS Fatigue, the sensitivity was 0.448 and specificity was 0.791 with a 0.664 area under the curve (AUC) value, which is interpreted as an acceptable discrimination ability [36]. In the case of SF36-MCS sensitivity was 0.421, and specificity was 0.770, with a 0.625 AUC value. During the measurement of the MCID for improvement in FACIT-FS, varied results were shown according to VAS Fatigue, in which 4 points represented an improvement, and using the SF36-MCS as an anchor, it was 2 points. It was determined to allocate 4 points regarding improvement. Sensitivity with VAS was 0.381 and regarding SF36-MCS it was 0.314. Specificity was 0.814 with VAS Fatigue and 0.824 with SF36-MCS. The performed ROC curve analyses to examine the discriminative performance for improvement resulted in AUC of 0.660 and 0.608, which are acceptable [36] (Figure 1). To confirm the validity of these results, post hoc power calculations were performed. In the case of VAS Fatigue, the empirical power was found to be 0.943 and 0.963 regarding worsening and improvement. In the case of SF36-MCS, these were found to be 0.747 and 0.844. Based on these, the calculated MCID values can be considered expressive.

Out of the 144 validated FACIT-FS questionnaires, patients were categorized according to the MCID value. Forty-four patients’ fatigue worsened during the follow-up period, 35 patients improved, while 65 remained unchanged.

**Figure 2:**
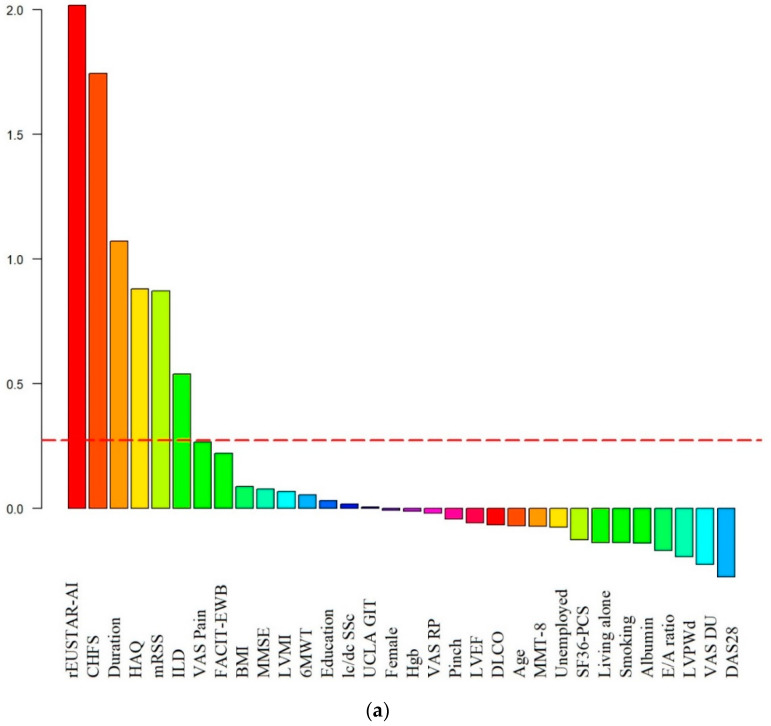

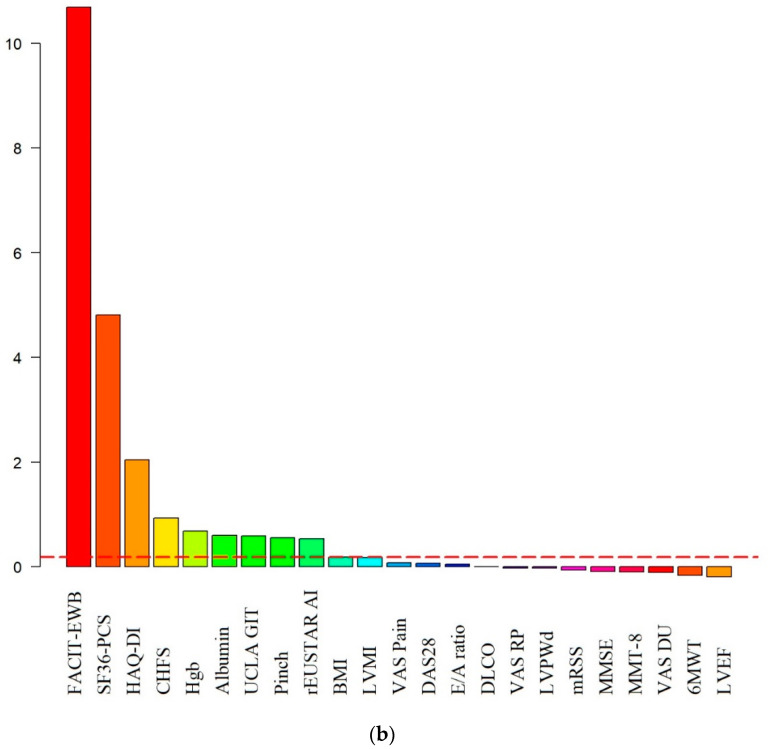
Results of the Random Forest algorithm for the FACIT-FS changes to select the most important factors regarding alterations in levels of fatigue during 12-month follow-up in 144 followed patients with systemic sclerosis. (**a**) Potential predictors among the baseline parameters; (**b**) Influencing factors of the changes of the results FACIT -FS during the follow-up. Y axis represents the relative variable importance (Arbitrary unit). All variables beyond the red dashed line are considered as potential predictors. rEUSTAR AI, revised European Scleroderma Trials and Research Group Activity Index; CHFS, Cochin Hand Function Scale; Duration, Disease Duration Time; HAQ, Health Assessment Questionnaire Disability Index; mRSS, Modified Rodnan Skin Score; ILD, Interstitial Lung Disease; VAS, Visual Analogue Scale; FACIT-EWB, The Functional Assessment of Chronic Illness Therapy Emotional Well-Being; BMI, Body Mass Index; MMSE, Mini Mental State Examination; LVMI, Left Ventricular Mass Index; 6MWT, Six-minute Walk Test; lcSSc, limited cutaneous Systemic sclerosis; dcSSc, diffuse cutaneous Systemic sclerosis; UCLA GIT, University of California, Los Angeles Scleroderma Clinical Trial Consortium Gastrointestinal Tract Instrument 2.0; Hgb, Hemoglobin; RP VAS, VAS for Raynaud’s Phenomena; Pinch, Pinch Strenght; LVEF, Left Ventricular Ejection Fraction; DLCO, Diffusing Capacity for Carbon Monoxide; MMT8; Manual Muscle Testing-8; DU VAS, VAS for Digital Ulcers; DAS28, Disease Activity Score 28; SF36-PCS, Short Form 36 Physical Component Summary; LVPWd, End-Diastolic Left Ventricular Posterior Wall thickness.

Two approaches were used to predict changes in fatigue perception. First, during the identification of independent predictors, the baseline parameters were examined, secondly these parameters’ changes during the follow-up period. Since the numerous parameters investigated, the RF algorithm was used to select the most important factors. The potentially relevant variables according to RF from the baseline parameters were sequentially in order: the rEUSTAR-AI, the CHFS, disease duration, HAQ-DI, MRSS and the presence of ILD. In the case of changes in parameters over a one-year span, the selected variables were FACIT-EWB, SF36-PCS, HAQ-DI, the CHFS, Hgb levels, albumin levels, the UCLA GIT 2.0, pinch strength and the rEUSTAR-AI. The variables considered above were selected for the binary logistic regression procedure (Figure 2).

During binary logistic regression analyses, HAQ-DI was omitted among the selected variables in every examination to prevent multicollinearity, since HAQ-DI was strongly correlated with CHFS. When examining the improved group versus the worsened and unchanged, disease duration (OR: 0.92, CI: 0.846–0.979, *p* = 0.020), the CHFS (OR: 1.03, CI: 1.003–1.073, *p* = 0.030) and the rEUSTAR-AI (OR: 1.37, CI: 1.034–1.850, *p* = 0.030) proved to be significant independent predictors among the baseline data (Table 4).

In examining the changes of parameters, the increasing FACIT-EWB (OR: 1.3, CI: 1.157–1.593, *p* < 0.001) and SF36-PCS (OR: 1.05, CI: 1.003–1.094, *p* = 0.040) proved to be significant independent predictors of improvement, however, increasing UCLA GIT 2.0 (OR: 0.08; CI: 0.007–0.616, *p* = 0.020) scores lowered the chance to be in the improved group during the follow-up period. In the examination of the worsened vs. the improved and unchanged, only the presence of ILD predicted the worsening in levels of fatigue (OR: 3.2 CI: 1.247–9.158; *p* = 0.020) from baseline data. Increasing FACIT-EWB (OR: 0.87, CI: 0.776–0.962, *p* = 0.009) and SF36-PCS (OR: 0.95, CI: 0.916–0.982 *p* = 0.004) resulted in a lower chance to be in the worsened fatigue group during the second examination (Table 4).

Examining the changes of rEUSTAR-AI among patients who improved regarding fatigue, showed a significant decreasing tendency of activity from 3.1 (2.1; 4.6) to 2.4 (1.25; 3.9) points (*p* = 0.43). In the worsening fatigue group activity scores varied from 1.6 (1.0; 3.4) at baseline to a 2.2 (1.3; 3.4) value at follow-up (*p* = 0.694).

## 4. Discussion

In this one-year follow-up observational study, we demonstrated fatigue symptoms in patients with SSc developed early in the onset of the disease, and were strongly associated with their HRQoL, emotional disorders, pain, their functionalities and physical conditions. The levels of fatigue were monitored using different PROMs. Notably, for the first time, the MCID values were determined for FACIT-FS among scleroderma patients. The most important predictors of improvement in levels of fatigue from baseline parameters were the significant disease activity, the patients’ poorer functionality and the shorter disease duration. However, SSc patients with ILD during the first examination had approximately tripled risks for worsening fatigue. The most influential independent factors regarding the changing of FACIT-FS during the follow-up were improving or worsening in the same direction, in reference to physical condition, GI symptoms and emotional factors.

### 4.1. Baseline Results

We found FACIT-FS scores 38.5 (29; 45.3) among SSc patients in our sample, which is similar to the results of Strickland et al., in which it was 35 (27.5; 44.0) among 68 patients [5]. In another sample, lower scores were apparent: 32.2 ± 12.1 among 785 patients [11], however, the patients’ mean age was higher and their mean duration was longer in this particular study. There was another observation, in which the median Fatigue-FS score was 31.0 (20.0; 41.0) in a sample of 326 early dcSSc patients [10] with significantly more severe organ involvements and disease activity. In our investigation patients with higher disease activities also suffered from more severe fatigue (Table 3), when compared to individuals without significant disease activity. Similarly, in other chronic systemic autoimmune diseases, in which disease activities are more frequently characteristic, fatigue is usually more predominant, as in other observations, with 348 patients afflicted with systemic lupus erythematosus (SLE) (25.7 ± 12.0) [37] or with 271 rheumatoid arthritis (RA) (29.17 ± 11.06) [16]. 

Notably, fatigue levels did not significantly differ between the early- and late-phase SSc patients, indicating chronic fatigue is one of the early onset symptoms during the disease course. In early phases of the disease fatigue may be associated with early microvascular changes with endothelial cell dysfunction, followed by autoimmune inflammation and hypoxia, while in late-phases general vasculopathy and fibrosis cause significant hypoxia, resulting in the experience of fatigue. 

Fatigue is a multi-dimensional symptom with physical, emotional, cognitive, and social aspects, diminishing HRQoL [38]. In this study, correlation examinations revealed the most intense associations of exhaustion with HRQoL, pain and emotional and physical conditions (Table 2). Generally, in chronic diseases, HRQoL is diminished. It can be simply measured and monitored using PROMs. In the current study, HRQoL was assessed using the SF-36 MCS, PCS and FACIT-F, which strongly correlated with all kinds of fatigue measure results. In previous studies [10,39] PROMs, regarding the aspects of HRQoL also correlated strongly with fatigue levels, drawing attention, in which the management of fatigue may help improve the quality of patients’ lives.

In chronic rheumatic diseases, most patients have different origins of pain. The most common sources of pain in SSc include joint pain, joint contractures, digital ulcers, Raynaud’s phenomenon and GI symptoms [40,41]. In our study, we also found significant correlations with these painful organ manifestations, assessed by Raynaud’s VAS, active polyarthritis measured by DAS-28, and GI symptoms using UCLA SCTC GIT 2.0 (Table 2). A study examining the pain and itch symptoms affecting SSc patients, showed pain is associated with poorer HRQoL and sleep quality, and more fatigue, emphasizing the complexity of this relationship [42].

Similar to earlier studies [13,43], our results confirmed the importance of mood disorders, cognitive and social factors, since our patients, who suffered from loneliness, unemployment, depression and/or anxiety experienced increased levels of fatigue (Table 3). In our study cognitive function measured with MMSE also indicated mild deterioration and association with fatigue. Moreover, in our sample, sleep disturbances, and exhaustion which are connected closely to emotional health [12], were significantly more frequent in SSc patients, when compared to individuals without SSc (Table 1). 

Correlation examinations revealed an intense association of exhaustion with physical performance measured with the 6MWT, MMT-8 and pinch strength. 

6MWT in SSc is associated with pulmonary fibrosis, PAH, and cardiac disease [44]. However, in a study conducted with SSc patients without evidence of heart and lung involvement, patients still had a lower aerobic capacity, when compared to controls [45]. Current examinations [45,46] showed associations between 6MWT and muscle strength, suggesting vascular damage causing muscle hypoxia may play a role in exercise intolerance resulting in the experience of fatigue. Muscle involvement in SSc is a frequent complication, up to 90% of patients may have muscle weakness, resulting from several possible etiologies [47]. We found muscle weakness also has a significant impact upon fatigue perception (Table 2). The beneficial effect of improving physical condition including muscle strength is supported by previous studies [48,49], in which SSc patients, who participated in a complex training program, achieved improvements regarding many aspects of their conditions, including fatigue. 

Additionally, loss of ability, based on HAQ-DI and CHFS tests had a strong correlation with fatigue levels. Several conditions may have an impact upon hand functionality in SSc, such as polyarthritis, contractures, sclerodactyly, tendon friction rubs, subcutaneous calcinosis, ischemia, or muscle weakness. These symptoms may interfere with the daily routine of patients [50,51], prolonging the time to finish simple tasks, which may be distressing and exhausting both mentally and physically. 

Among our patients with SSc, females reported higher levels of fatigue when compared to the males, however, male patients screened were significantly younger. In a longitudinal study, examining 225 SSc patients, female gender was found to be a significant predictor of higher fatigue levels [52]. Limited and diffuse SSc patients’ suffered from similar levels of fatigue, which is parallel to what Murphy et al. found in their sample [43]. Generally, patients with dcSSc have poorer conditions because of higher disease activity and more severe organ involvements, when compared to lcSSc. However, in our sample, lcSSc individuals had a higher average age, a longer disease duration and they also had worse social parameters. 

The levels of exhaustion between the different serological subgroups proved to be the same, even among the groups with “ACA”, “anti-Topo I” or “anti-RNAP3”, “only ANA”, or “without these specified autoantibodies” (Figure S1 in Supplementary Material), implying age, disease duration, social aspects, and other disease-related characteristics are likely more relevant regarding this symptom. However, in a longitudinal study [53], the presence of anti-U1-RNP antibodies was identified as having a negative impact upon fatigue perception. Admittedly, in this study anti-U1-RNP antibodies were not investigated. 

According to the presence or absence of different internal organ manifestations, those who had GI and/or pulmonary involvements experienced diminished levels of vitality, however, lung disease was miniscule when considering the threshold of significance (Table 3). Interestingly, in this cohort, neither FVC nor DLCO of the respiratory function parameters were correlated with exhaustion at the baseline, nor in follow-up investigations. Other studies have described fatigue’s correlation with FVC and DLCO [10], however, in our sample, these associations could not be confirmed. Regarding cardiac involvement, the correlations of fatigue were mainly observed in the presence of diastolic disorder, as it is an important characteristic in this disease [30]. In addition, left ventricular hypertrophy (LMVI) also indicated a significant correlation with fatigue. According to Peytrignet et al. [10] 326 SSc patients with cardiac involvement also showed significantly higher levels of fatigue based on FACIT-FS.

### 4.2. Results of the Follow-Up

During the follow-up, no significant changes were detectable in patients’ fatigue levels, aligned with the findings of Assasi et al. and Willems et al., who also reported levels of exhaustion stabilized over the follow-up period [52,53]. Individual fluctuations were present in our sample, however, in the entire cohort it resulted in the same FACIT-FS median value at follow-up. In detecting individual changes, the MCID value was determined for FACIT-FS, and it was the first time when MCID for FACIT- FS in SSc was calculated. In this sample of patients, deterioration of fatigue was −3 points, while improvement was 4 points. Based on the post hoc power calculation, these results represent valid values. Similar results were reported in other published studies conducted with patients suffering from systemic autoimmune diseases, such as RA [16], and SLE [54], in which the MCID value for FACIT-FS was 3–4 points and 3–6 points. Additionally, it was previously described, the MCID values of FACIT scales are seemingly relatively stable among different patient populations [55], and our results also support this finding. Using MCID values is important in monitoring changes and for the interpretation of the results of the PROMs either in scientific works or in daily practice. 

The independent predictors of fatigue alterations include the disease duration, the presence of ILD, the CHFS and the rEUSTAR-AI from baseline parameters, based on binary logistic regression. 

Strikingly, patients with longer disease duration had a poorer chance to improve regarding fatigue during the follow-up compared to individuals with early phase SSc. This might be due to high disease activity, which is predominant in early cases, causing inflammation, hypoxia and fatigue, which can be treated more successfully, than in late-phases SSc, when there are mostly irreversible changes, tissue fibrosis and atrophy [56]. 

Notably, suffering from ILD was a significant predictor of worsening fatigue, with a remarkable 3.2-fold odds ratio in our sample, parallel to the results of a longitudinal study, conducted by Assasi et al. [53], in which lower levels of baseline DLCO predicted fatigue impairment. Pulmonary fibrosis is a highly progressive frequent symptom in SSc, in which patients suffer from dyspnea and weakness. Inexplicably, pulmonary function test results did not show correlations with fatigue in our sample.

Interestingly, those who had higher disease activity scores at baseline tended to score lower levels of fatigue during the second examination. This implies that those who started from a worse condition, with more active disease, may improve more than those who began with a relatively stable condition or had been diagnosed with more damage than activity. We also find a significant improvement in activity scores among the patients who improved regarding fatigue. 

Patients who started with more severe disabilities using CHFS in our sample, were more likely to improve in reference to fatigue. However, we examined how both HAQ-DI and CHFS scores change during the follow-up, and found, individuals who had worse scores at baseline tended to improve regarding disabilities as well, and who started from a better condition, rather worsened, or remained stable. This is similar to the trend that Peytrignet et al. [10] found in their sample of early dcSSc patients, those who had more disabilities at baseline generally improved, and patients with the least disability more likely tended to worsen. 

In our cohort, patients with diminishing GI symptoms during the follow-up period were significantly less likely to have improved fatigue. Therefore, in this sample, the importance of GI symptoms has already been emphasized in three aspects: (1) the higher degree of fatigue in the GI-affected group, (2) the UCLA-GIT test strong correlation to the level of fatigue, and (3) the independent predictive role of GI symptoms’ deterioration in feeling more exhausted. This confirms the results of previous studies, in which GI symptoms were strongly associated with fatigue, and were also predictors of exhaustion [11,53]. SSc can affect every part of the GI tract and presents as a variety of symptoms. The UCLA GIT SCTC 2.0 questionnaire, which was used in this study, contains the entire possible spectrum of symptoms and may result in high individual variance. A more detailed assessment of GI symptoms will be necessary to draw accurate conclusions. For example, malnutrition as the result of malabsorption may exacerbate fatigue, however, reflux symptoms, occurring in a large proportion of patients also proved to be a predictor of sleeping disturbances [57], which may lead to exhaustion. The last-mentioned fact may support the finding of a study, in which the absence of upper GI symptoms predicted less fatigue [5].

The improvement in emotional and physical well-being were associated with decreasing fatigue, and the deterioration of these parameters increased the risk of worsening fatigue during the follow-up. In a cross-sectional study of 752 SSc patients [58], performing regular physical exercises was associated not only regarding vitality but also lower symptoms of anxiety and depression.

The main limitations of the current study are the relatively small sample size, the lack of adequate examination of individuals without SSc at the second investigation, and the short follow-up period. In our tertiary centre, we are primarily focused on severe cases, although we treat a wide swath of diseases, hence, mild cases may not be fully represented. From a varied perspective over a span of 12 months, the levels of fatigue may not change remarkably. A key strength of the study is the diverse range of investigations performed.

## 5. Conclusions

In summary, our study confirmed fatigue affecting patients with SSc was significantly more severe when compared to individuals without SSc. Fatigue is an early-onset symptom during the disease course, however, patients with shorter disease duration had even more chance to improve, when compared to late-phase patients in our sample. Fatigue is a complex, multifactorial symptom, most strongly correlated with HRQoL, the levels of pain, mood disorders, physical condition and functionality. By defining MCID values of the FACIT-FS fatigue can be followed more effectively both in clinical trials and in shared decisions with patients in daily practice. Using multivariable regression analysis, ILD, disease activity, SSc duration time, hand functionality, physical and emotional well-being all were independent predicting alterations in levels of fatigue. From a clinical perspective, this study identified factors, which are potentially modifiable and may help in managing fatigue. Considering our outcomes, we conclude patients suffering from SSc who presented diminished levels of physical condition also reported an increase in severe fatigue. Thus, early regular physical training may prove effective against the development of fatigue symptoms in patients with SSc.

## Figures and Tables

**Table 1:** Clinical and demographical characteristics of 160 patients afflicted with Systemic Sclerosis (SSc) and 62 non-SSc-suffering control individuals.

Investigated Factors	SSc *n* = 160	Non-SSc-Suffering Controls *n* = 62	*p*
Females	138 (86.3%)	49 (79.0%)	0.186
Age, years	55.8 ± 13.0	54.6 ± 13.7	0.411
Disease duration, years	10.5 ± 7.6	-	-
Diffuse cutaneous SSc	88 (55%)	-	-
Body Mass Index, kg/m^2^	25.8 ± 5.1	27.1 ± 4.4	0.326
Living alone	67 (41.9%)	29 (46.8%)	0.596
Employed	50 (31.3%)	37 (59.7%)	<0.001
Sleeping disorder	79 (49.4%)	17 (27.41%)	0.008
Modified Rodnan Skin Score >14	22 (13.75%)	-	-
Revised EUSTAR Activity Index	2.5 (1.3; 3.9)	-	-
Pulmonary fibrosis, proven with HRCT	97 (60.6%)	-	-
Forced Vital Capacity, %	104.7 (88.0; 114.0)	118 (105.0; 129.0)	<0.001
Diffusing Capacity for Carbon Monoxide, %	66 (51.0; 77.0)	87 (80.5; 95.0)	<0.001
Cardiac involvement	114 (71.3%)	-	-
E-A Ratio	1 (0.8; 1.3)	-	-
Diastolic dysfunction	107 (66.9%)	-	-
Left Ventricular Mass Index (LVMI)	100 (88.7; 112.4)	-	-
Six-minute Walk Test, m	428 (347; 523)	600 (510; 660)	<0.001
GI involvement	47 (29.4%)	-	-
UCLA SCTC Gastrointestinal tract 2.0	0.2 (0.1; 0.5)	0.04 (0; 0.2)	<0.001
Hemoglobin, g/L	130 (124.0; 139.8)	142 (130.3; 147.8)	<0.001
Albumin, g/L	43.5 (41.6; 46.0)	48 (45.4; 49.7)	<0.001
Disease Activity Score 28	2.7 (20; 3.8)	1.7 (1.3; 2.3)	<0.001
Health Assessment Questionnaire, (0–3)	0.75 (0; 1.38)	0 (0; 0)	<0.001
Manual Muscle Testing-8 (0–150)	122.0 (110.0; 133.0)	135 (122.5; 143.5)	<0.001
Pinch strength, N	61.7 (46.7; 75)	71.6 (60.8; 83.3)	<0.001
Chochin Hand Function Scale, (0–90)	5(0; 17.0)	0 (0; 0)	<0.001
Absence of major autoantibodies	28 (17.5%)	-	-
Only anti-nuclear antibodies	39 (24.4%)	-	-
Anti-DNA topoisomerase I	41 (25.6%)	-	-
Anti-centromere antibodies	32 (20.0%)	-	-
Anti-RNA Polymerase III	16 (10.0%)	-	-
Absence of major autoantibodies	28 (17.5%)	-	-
Mini Mental State Examination (0–30)	28 (27; 29)	29 (28; 29)	0.014
VAS Digital Ulcer (0–100)	0 (0; 7.5)	-	-
VAS Raynaud’s (0–100)	18.5 (0.3; 45.0)	-	-
VAS Pain (0–100)	19 (2; 50)	0 (0; 1)	<0.001
VAS Fatigue (0–100)	24 (3; 56.3)	0.00 (0; 5)	<0.001
SF36-Vitality (0–100)	51.5 (40; 75)	75 (65; 85)	<0.001
FACIT-Fatigue Scale (0–52)	38.5 (29; 45.3)	48.0 (44; 50)	<0.001
FACIT-Emotional Well-being (0–24)	19 (16; 22)	21 (19; 23)	<0.001
FACIT-F (0–160)	115.2 (90; 133.4)	141 (135; 151.5)	<0.001
SF36-Mental Component Summary (0–100)	68.1 (40.3; 83.8)	85.7 (74.3; 94.3)	<0.001

Values are depicted as median (first quartile; third quartile) or mean ± standard deviation. Qualitative values are represented in numbers of cases and percentages. EUSTAR, European Scleroderma Trials and Research Group; UCLA SCTC GIT 2.0, University of California, Los Angeles Scleroderma Clinical Trial Consortium Gastrointestinal Tract Instrument 2.0; VAS, Visual Analogue Scale; SF36, Short Form 36; FACIT, The Functional Assessment of Chronic Illness Therapy.

**Table 2:** Correlations between baseline parameters and fatigue measures among 160 SSc patients.

	Spearman’s Correlation Coefficients		
Clinical Data	VAS Fatigue	SF36-Vitality	FACIT-Fatigue Scale
Age, years	0.202 *	−0.268 **	−0.233 **
Disease duration, years	0.167 *	−0.058	−0.016
Body Mass Index, kg/m^2^	0.072	−0.107	−0.146
Modified Rodnan Skin Score (only in dcSSc, *n* = 88)	−0.305 **	0.242 *	0.193
Disease Activity Score 28	0.523 **	−0.426 **	−0.503 **
Health Assessment Questionnaire Disability Index	0.571 **	−0.565 **	−0.679 **
Revised EUSTAR Activity Index	0.145	−0.113	−0.177 *
Cochin Hand Function Scale	0.573 **	−0.509 **	−0.640 **
Left Ventricular Ejection Fraction, %	−0.013	0.075	0.007
Left Ventricular Mass Index	0.309 **	−0.329 **	−0.365 **
Diastolic dysfunction, E-A Ratio	−0.181 *	0.197 *	0.220 **
Six-minute Walk Test, m	−0.394 **	0.439 **	0.444 **
Pinch strength, N	−0.355 **	0.322 **	0.455 **
Manual Muscle Testing-8	−0.391 **	0.420 **	0.458 **
Forced Vital Capacity, %	0.103	−0.051	−0.006
Diffusing Capacity for Carbon Monoxide. %	−0.099	0.115	0.104
UCLA SCTC Gastrointestinal test 2.0	0.387 **	−0.456 **	−0.495 **
Mini Mental State Examination	−0.262 **	0.234 **	0.201 *
VAS Raynaud’s	0.564 **	−0.429 **	−0.454 **
VAS Pain	0.707 **	−0.515 **	−0.577 **
Short Form 36-Physical Component Summary	−0.677 **	0.713 **	0.776 **
Short Form 36-Mental Component Summary	−0.647 **	0.885 **	0.807 **
FACIT- Emotional Well-being	−0.397 **	0.583 **	0.671 **

Assessed with Spearman’s rank correlations * *p* < 0.05; ** *p* < 0.01; VAS, Visual Analogue Scale; FACIT, The Functional Assessment of Chronic Illness Therapy; EUSTAR, European Scleroderma Trials and Research Group.

**Table 3:** Comparing fatigue symptoms based on the results of the Functional Assessment of Chronic Illness Therapy-Fatigue Scale (FACIT-FS) between different subgroups of 160 patients with systemic sclerosis (SSc).

Factors	Presence	Absence	
	Results of FACIT- FS		*p*-Values
Diffuse cutaneous SSc	41.0 (29.5; 48.0)	36.0 (28.0; 44.0)	0.092
<4 years SSc duration (early SSc)	40.0 (28.2; 45.0)	38.5 (30.0; 46.0)	0.501
Disease activity (rEUSTAR AI > 2.5)	36.0 (27.0; 43.0)	40.5 (32.5; 48.0)	0.033
UCLA GIT 2.0 > 1	16.5 (11.0; 23.875)	40.0 (32.25; 47.5)	<0.001
Pulmonary involvement, FVC < 80%, DLCO < 70%	37.5 (28.6; 43.0)	40.5 (30.0; 49.0)	0.05
Diastolic dysfunction	38.0 (29.0; 44.0)	45.0 (30.0; 50.0)	0.037
HAQ-DI > 1.0	32.0 (24.0; 37.0)	44.0 (37.0; 49.0)	<0.001
Visual Analogue Scale-Pain > 40	29.5 (22.0; 38.0)	42.0 (35.0; 49.0)	<0.001
Anxiety/depression (EQ-5D)	32.0 (25.0; 39.0)	44.0 (38.0; 50.0)	<0.001
Living alone	36.0 (28.0; 41.5)	42.0 (30.5; 47.5)	0.045
Unemployed patients of working age	36.0 (27.0; 43.5)	46.58 (34.0; 50.0)	0.001
Sleeping disorder	35.0 (24.0; 40.0)	44. (32.75; 50.0)	<0.001

Values are depicted as median (first quartile; third quartile); rEUSTAR AI, revised European Scleroderma Trials and Research Group Activity Index; UCLA GIT 2.0, University of California, Los Angeles Scleroderma Clinical Trial Consortium Gastrointestinal Tract Instrument 2.0; HAQ-DI, Health Assessment Questionnaire Disability Index; EQ-5D, Euro Quality of Life-5Dimension.

**Table 4:** Predictors of alterations in levels of fatigue during 12-month follow-up selected with Random Forest algorithm (Figure 2) in 144 followed (44 worsened, 35 improved, 65 unchanged regarding fatigue) patients with systemic sclerosis using binary logistic regression analyses.

		Worsening (*n* = 44)			Improvement (*n* = 35)		
		Odds Ratio	CI	*p*	Odds Ratio	CI	*p*
	Predictors of Worsening/Improvement Fatigue among Baseline Parameters.						
Based on baseline data	rEUSTAR AI	0.825	0.616–1.087	0.183	**1.374**	1.034–1.850	**0.030**
	Cochin Hand Function Scale	0.982	0.949–1.014	0.289	**1.037**	1.003–1.073	**0.031**
	Disease duration	1.027	0.969–1.086	0.356	**0.916**	0.846–0.979	**0.018**
	Modified Rodnan Skin Score	0.901	0.806–0.995	0.052	0.924	0.837–1.011	0.101
	Interstitial Lung Disease	**3.197**	1.247–9.158	**0.021**	0.602	0.238–1.541	0.284
	Factors, which changes predict the worsening/improvement in fatigue levels.						
1-year changing values	FACIT-EWB	**0.868**	0.776–0.962	**0.009**	**1.338**	1.157–1.593	**<0.001**
	SF36-PCS	**0.951**	0.916–0.982	**0.004**	**1.045**	1.003–1.094	**0.042**
	Cochin Hand Function Scale	1.020	0.946–1.100	0.590	1.006	0.923–1.082	0.876
	Hemoglobin	0.971	0.923–1.015	0.215	1.032	0.973–1.095	0.267
	Albumin	0.937	0.809–1.081	0.380	1.187	0.988–1.451	0.075
	UCLA SCTC GIT 2.0	1.953	0.383–10.478	0.422	**0.077**	0.007–0.616	**0.020**
	Pinch strength	1.004	0.967–1.041	0.822	1.043	0.999–1.091	0.055
	rEUSTAR AI	0.937	0.720–1.213	0.622	1.120	0.821–1.558	0.482

Statistically significant results are indicated in bold. CI, Confidence Interval; rEUSTAR AI, revised European Scleroderma Trials and Research Group Activity Index; FACIT-EWB, The Functional Assessment of Chronic Illness Therapy Emotional Well-being; SF36-PCS, Short Form 36 Physical Component Summary; GIT 2.0, University of California, Los Angeles Scleroderma Clinical Trial Consortium Gastrointestinal Tract Instrument 2.0.

## Data Availability

Data are not publicly available due to ethical issues.

## Supplementary Materials

The following are available online at https://www.mdpi.com/article/10.3390/ijerph20010771/s1. Figure S1: The scores of The Functional Assessment of Chronic Illness Therapy Fatigue Scale (FACIT-FS) in different patient groups according to the present autoantibodies in 157 with Systemic sclerosis.
