# Supplementary material for: Minimal Clinically Important Differences (MCID) for the Functional Assessment of Chronic Illness Therapy Fatigue Scale in Patients with Systemic Sclerosis
Source: Int J Environ Res Public Health. 2022 Dec 31;20(1):771. doi: 10.3390/ijerph20010771 (PMC9819291; doi:10.3390/ijerph20010771)
Supplement: Supplementary file 1 [file ijerph-20-00771-s001.zip › ijerph-2102161-supplementary.pdf]

Supplementary Material

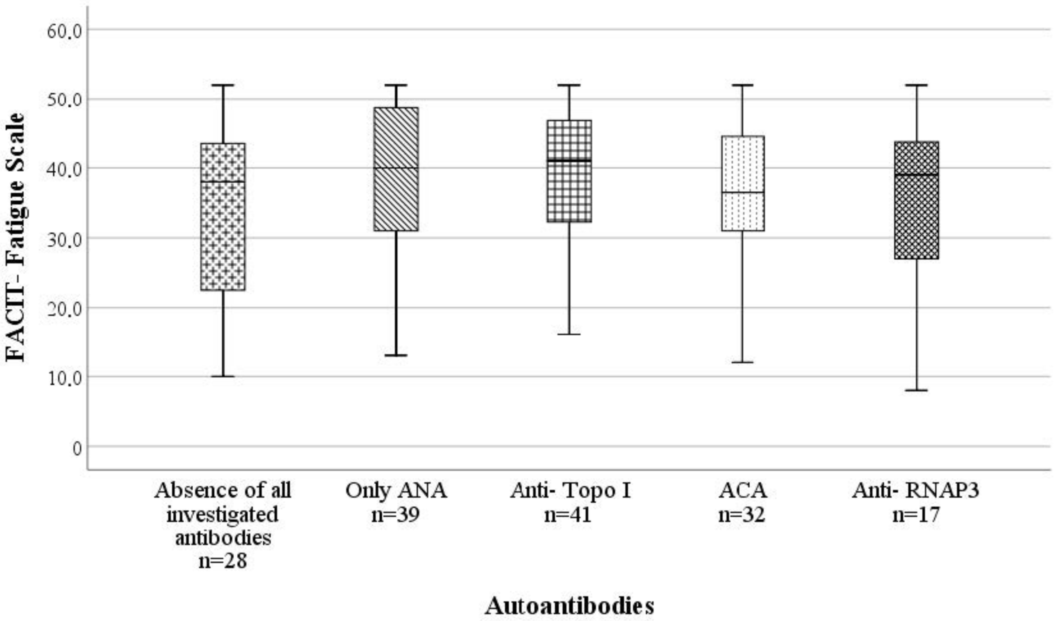

Figure S1. The scores of The Functional Assessment of Chronic Illness Therapy Fatigue Scale (FACIT-FS) in different patient groups according to the present autoantibodies in 157 SSc cases.

ANA, Anti- nuclear antibodies; Anti- Topo I, Anti- DNA topoisomerase I; ACA, Anti-centromere antibodies; anti- RNAP3, Anti – RNA Polymerase III.
